# It Is Premature to Use Postmortem Sperm for Reproductive Purposes: a Data-Driven Opinion

**DOI:** 10.1007/s43032-022-00874-6

**Published:** 2022-02-10

**Authors:** Juan J. Tarín, Miguel A. García-Pérez, Antonio Cano

**Affiliations:** 1 Department of Cellular Biology, Functional Biology and Physical Anthropology, Faculty of Biological Sciences, University of Valencia, Dr. Moliner 50, 46100 Burjassot, Valencia Spain; 2 Institute of Health Research INCLIVA, Valencia, Spain; 3 Department of Genetics, Faculty of Biological Sciences, University of Valencia, Dr. Moliner 50, 46100 Burjassot, Valencia Spain; 4 Service of Obstetrics and Gynecology, University Clinic Hospital, Valencia, Spain; 5 Department of Pediatrics, Obstetrics and Gynecology, Faculty of Medicine, University of Valencia, Av. Blasco Ibañez 17, 46010 Valencia, Spain

**Keywords:** Assisted reproduction technology, Coding and non-coding RNAs, Epigenetic changes, Interval from death to sperm retrieval, Offspring, Postmortem protein, DNA, and RNAs degradation

## Abstract

Postmortem sperm retrieval for reproductive purposes is an assisted reproduction procedure that offers women an opportunity to have a child using sperm retrieved from their deceased partners. The ethical issues of this procedure have been discussed in previous works. However, an assessment of the procedure using a scientific perspective is still lacking. Here, we aim to ascertain, using a biological standpoint, whether postmortem sperm should be rescued for reproductive purposes. Data suggest that it is premature to use postmortem sperm for reproductive purposes. This procedure should not be clinically applied until appropriate and comprehensive analyses have been completed. Such analyses should be focused not only on fertilization, embryo development, and pregnancy outcomes, but also on potential postmortem alterations of sperm DNA, RNAs, and proteins. In addition, genetic and epigenetic analyses of sperm, pre-implantation embryos, and newborns, as well as mental and physical health follow-up of the resulting offspring during a whole life cycle, using appropriate non-human mammalian models, are warranted.

## Introduction

The safety of assisted reproduction techniques (ARTs) is usually assessed *after* (not *before*) ARTs are incorporated into a clinical setting. The common end-points typically analyzed in clinical trials and less frequently in randomized controlled trials are fertilization, pre- and post-implantation embryo development, obstetric and perinatal outcomes, and follow-up of the resulting offspring during infancy, childhood, and adolescence. However, *before* incorporating a new ART into clinical practice, other cellular, molecular, genetic, and epigenetic aspects should be assessed using adequate laboratory and appropriate animal experimentation [1]. Unfortunately, literature shows that this is not the case on most occasions [2–6]. The use of postmortem sperm for reproductive purposes is an example of this praxis.

ART stressors such as gamete/embryo cryopreservation, oocyte in vitro maturation, intracytoplasmic sperm injection (ICSI), in vitro culture system, and embryo biopsy [7], as well as gamete/embryo exposure to suboptimal ART conditions [8], may induce genetic and epigenetic changes in gametes and/or pre-implantation embryos. Likewise, gamete and offspring genetic and epigenetic disturbances may be associated with parental exposure to environmental and occupational xenobiotics [7]. Sperm exposure to the altered body fluids and/or degenerating tissues of dead men may also pose a genetic and/or epigenetic risk for spermatozoa and prospective offspring. Unfortunately, as far as we know, these potential effects have not yet been assessed in men or males of non-human mammalian species.

In the present study, we aim to gather data to support or reject the clinical use of postmortem sperm for reproductive purposes. The study is exclusively focused on biological and clinical data. Readers interested in ethical questions and legal and religious implications of this procedure are referred to several excellent papers dealing with these topics [9–13].

## Methods

In order to analyze all the live birth cases reported to date, a literature search was performed to identify all the relevant publications recorded on PubMed from database inception up to October 2021 using the following key words: posthumous/postmortem sperm retrieval, posthumous/postmortem human reproduction, and interval from death to sperm retrieval. References cited in relevant articles were also examined. The literature search was exclusively focused on clinical deaths, not on brain deaths of men on ventilatory support.

## Live Births Reported to Date

Table 1 displays the men’s age, cause of death, sperm retrieval schedule, and sperm freezing methodology reported in seven live birth cases of women that underwent standard in vitro fertilization (IVF) and/or ICSI cycles using testicular or epididymal spermatozoa of their deceased husbands. The details of the thirteen oocyte retrieval cycles undergone by these women are shown in Table 2.

**Table 1:** Reported data of deceased men whose postmortem testicular or epididymal spermatozoa were used for reproductive purposes

Case	Man’s age (yrs)	Cause of death	Interval (h) between death and sperm retrieval	Source of spermatozoa	Sperm freezing
1 [9, 14]	*	Sudden allergic reaction	30	*	*
2 [15]	*	*	30	Epididymides	*
3 [16, 17]	38	Heart attack	3	Testes	Liquid nitrogen vapor
4 [18]	*	Motor vehicle accident	48	Testes	Slow freezing
5 [18]	39	Ischemic heart disease	12	Testes	Slow freezing
6 [18]	39	Workplace accident	23	Testes	Closed vitrification system
7 [18]	29	Motor vehicle accident	24	Testes	Closed vitrification system

*Data is not available

**Table 2:** Reported data of live births in women that underwent IVF and/or ICSI cycles using testicular or epididymal spermatozoa retrieved from their deceased husbands

Case	N° of oocyte retrieval cycle	Woman’s age (yrs)	Insemination technique	Spermatozoa injected	Fertilization (%)	Frozen embryos (%)	Transfer cycle (fresh/frozen)	N° of embryos transferred (fresh/frozen)	Pregnancy and short-term child health
1 [9, 14]	*`	*	ICSI	*	*	*	0/1	*	Female baby
2 [15]	*	*	IVF and ICSI	*	*	*	0/1	*	Healthy female baby
3 [16, 17]	1	36	ICSI	Immotile	6/15 (40.0)	2/4 (50.0)	1/1	2/2	Chemical pregnancy from the frozen cycle
	2	36	ICSI	Immotile	5/19 (26.3)	5/5 (100.0)	0/1	0/4	No
	3	37	ICSI	Immotile	10/19 (52.6)	--	1/0	3/0	No
	4	37	ICSI	Motile	6/13 (46.2)	0/3 (0.0)	1/0	3/0	No
	5	38	ICSI	Motile	4/14 (28.6)	0/0 (0.0)	1/0	4/0	Full term healthy baby
4 [18]	1	31	ICSI	Non-progressive motile sperm	5/10 (50.0)	3/4 (75.0)	1/0	1/0	Healthy female baby, 3.1 kg, 38 weeks of gestation (50th centile). Normal development up to one year of age
5 [18]	1	38	ICSI	Motile	8/20 (40.0)	2/7 (29.6)	1/0	1/0	Healthy female baby, 2.85 kg, 40 weeks gestation (between the 5th and 10th centile, as her older sibling conceived naturally). Normal development up to 5 year of age
6 [18]	1	41	ICSI	Motile	8/8 (100.0)	0/6 (0.0)	1/0	2/0	Preterm male infant, 1.63 kg, 31 weeks gestation (50th centile) and hospital discharge at 39 corrected weeks of gestation. Normal development up to 4 years of age, with close pediatric surveillance.
7 [18]	1	38	ICSI	*	*	*	*	*	Unsuccessful IVF cycle with low oocyte numbers and poor quality embryos
	2	38	ICSI	*	*	*	*	*	Unsuccessful IVF cycle with low oocyte numbers and poor quality embryos
	3	38	ICSI	Motile	5/5 (100.0)	0/3 (0.0)	1/0	2/0	Healthy female baby, 3.10 kg, 39 weeks gestation (25 centile). Normal development up to 7 year of age

*Data is not available

The first case described in the literature was a woman that gave birth to a baby girl using the sperm from her deceased husband, who died in 1995 as result of a sudden allergic reaction. This case was reported in the newspaper Los Angeles Times, in March 27, 1999 [14]. The sperm was harvested 30 hours after the husband’s death and frozen for 15 months before the woman’s oocytes were inseminated by ICSI [9, 14]. Likewise, Shefi et al. [15] informed of a case of a woman that gave birth to a healthy female child after undergoing standard IVF and ICSI using epididymal spermatozoa from her deceased husband. As in the live birth disclosed in the Los Angeles Times [14], the interval from death to sperm retrieval was 30 hours and further details of the case were not provided.

Note that the low number of cases and oocyte retrieval cycles undergone by the women analyzed in the present study prevents us from drawing solid conclusions about the potential effect of the interval between the man’s clinical death and sperm retrieval on embryology and pregnancy outcomes. Nevertheless, we have to point out that most of the babies born were girls. In particular, the reported sex ratio is 1:5 (i.e., 16.7% males versus 83.3% females) (in this analysis, we have excluded the full-term baby notified by Check et al. [16] since the sex of the newborn was not specified). Moreover, the four children recorded by Robson et al. [18] displayed an apparently normal development at least up to 1, 4, 5, and 7 years of age, respectively.

The low sex ratio observed, if confirmed after adding future cases, may be a consequence of the psychological disturbance and anxiety likely experienced by women after the death of their husbands, and/or the women’s excitement associated with having made the final decision to fertilize their oocytes using the sperm of their deceased husbands (we should bear in mind that “excitement” is a kind of stress called “eustress” that induces a moderate activation of the sympathetic nervous system, the hypothalamic-pituitary-adrenal axis, and the immune system [19]). Actually, maternal psychological stresses such as depression and anxiety, as well as experiencing stressful life events at the time of conception, are associated with drops in the number of male births in both humans and non-human mammals [20].

An alternative explanation for the preponderance of girls may lie on the fact that human Y spermatozoa under some sperm-storage conditions are more vulnerable to stressful environments than X spermatozoa [21], as may be encountered in the reproductive system of dead men. Nevertheless, we should take into consideration that no significant variation in the sex ratio of 18-day-old mouse fetuses generated after intracytoplasmic injection of cauda epididymis spermatozoa, retrieved from cadavers and maintained at 4°C from 0 to 20 days after death, has been reported [22]. These data suggest that the sex ratio may be unbiased when the number of cases analyzed increases.

## Data Against the Current Clinical Use of Postmortem Sperm for Reproductive Purposes

Shefi et al. [15] and Robson et al. [18] proposed to extend the upper 24-h limit of the interval from death to sperm retrieval, previously recommended by Tash et al. [23], to 36 and 48 h, respectively. Their proposal was based on the following facts: (1) Shefi et al. [15] observed that epididymal spermatozoa from a deceased man exhibited 5% motility and 7% viability (determined by vital stain) at 36 h after death; and (2) Robson et al. [18] found that testicular spermatozoa, displaying non-progressive motility at 48 h after death, were able to fertilize 5 out of 10 microinjected oocytes and contributed to the generation of a healthy baby girl. Their proposal, however, did not take into account other studies carried out in non-human mammalian species showing that the interval from death to sperm retrieval as well as the storage conditions, mainly temperature, for both the cadaver and sperm may affect sperm motility [24–29], sperm viability [22, 25, 27], fertilization [22], pre- and/or post-implantation embryo development [22, 24], and live birth incidence [27]. The source of spermatozoa (i.e., testes, epididymides, or vasa deferens) is another important factor we should bear in mind. For instance, at an ambient temperature of ≈ 22°C, degenerative changes in mouse testes are observed within 6 h after death. The alterations include disruption of intercellular bridges and sloughing of the germ cells into the lumen of the seminiferous tubules, and increased incidence of pyknotic nuclei in germ cells [24]. In contrast, postmortem degenerative changes in epididymides at the same ambient temperature are not clearly evidenced up to 12 h postmortem. On this occasion, the specific modifications observed in the epididymides are pyknosis of epithelial cells and release of their intracellular contents into the lumen [24].

Although animal models are essential to biological research, it may be argued that not all the results obtained on non-human mammalian species can be directly translated to humans [30]. However, even if we disregard the studies performed in animal models and assume a safe upper limit interval for human sperm retrieval between 24 [23] and 48 h [18], we could not be confident enough yet about the safety of this procedure. In fact, sperm motility and viability, fertilization, pre- and post-implantation embryo development, live birth incidence, and postnatal development from 1 to 7 years of age are not the only parameters that we should rely on to recommend the clinical use of postmortem sperm for reproductive purposes. Other lines of biological evidence demonstrating this procedure is harmless are needed, for instance, the assessment of the potential genetic and epigenetic anomalies in spermatozoa, pre-implantation embryos, and newborns, as well as short-, medium-, and long-term mental and physical health follow-up of the resulting offspring using appropriate animal models. In fact, although there may be differences among various tissues, organs, and genetic markers [31], postmortem degradation of DNA [32, 33] and RNA [31, 34] begins immediately after death because of endogenous nuclease activity and hydrolytic attack. Protein degradation also takes place after death although is slower and more reproducible than degradation of RNAs [35]. Moreover, we should remember that spermatids and spermatozoa may be transcriptionally and translationally active [36, 37]. They likely translate both nuclear and mitochondrial transcripts using mitochondrial ribosomes [36] and a stored heterogeneous population of sperm protein-coding messenger RNAs that may contribute to fertilization and early embryo development [36, 37]. Mature spermatozoa also store long non-coding RNAs (lncRNAs) and small non-coding regulatory RNAs (sncRNAs) including, among others, microRNAs, PIWI interacting RNAs, and transfer RNA-derived small RNAs [37, 38]. Notably, spermatozoa acquire fertilization and normal embryonic development capabilities after the sperm profiles of these lncRNAs and sncRNAs classes are remodeled during epididymal transit [37, 38]. That is to say, in order for spermatozoa to be functionally mature, they must interact with extracellular components secreted by epithelial cells lining the lumen of the epididymis, including not only proteins, DNA, lipids, ions, and nutrients but also lncRNAs and sncRNAs [37, 39].

Importantly, although microRNAs are less susceptible to degradation than other types of RNAs [40, 41], the sperm lncRNAs and sncRNAs profiles exhibit substantial plasticity and may be modified by paternal and sperm exposure to a variety of environmental conditions and stressful environments [37, 39]. Such epigenetic modifications may affect both early embryo development and the subsequent health of the offspring, including altered behavioral and metabolic phenotypes [37, 38]. Likewise, the stressful environment to which spermatozoa are exposed in the reproductive system of dead men may induce adverse long-term effects on offspring.

## Conclusion

Several lines of biological evidence suggest that postmortem sperm should not be rescued for reproductive purposes until appropriate and comprehensive analyses have been completed. Such analyses should be focused not only on fertilization, embryo development, and pregnancy outcomes, but also on potential postmortem alterations of sperm DNA, RNAs, and proteins. In addition, genetic and epigenetic analyses of sperm, pre-implantation embryos, and newborns, as well as mental and physical health follow-up of the resulting offspring during a whole life cycle, using appropriate non-human mammalian models, are warranted. These analyses should be performed even taking into account that the clinical demand for this ART procedure is relatively scarce.

## Data Availability

Not applicable.
